# Family caregivers’ sense-making of the results of functional neurodiagnostics for patients with Prolonged Disorders of Consciousness

**DOI:** 10.1080/09602011.2023.2299448

**Published:** 2024-01-17

**Authors:** Damian Cruse, Kotryna Ragazinskaite, Amy Chinner, Corinne Bareham, Neil Roberts, Ruth Banner, Srivas Chennu, Darrelle Villa

**Affiliations:** a Centre for Human Brain Health, University of Birmingham, Edgbaston, UK; b School of Psychology, University of Birmingham, Edgbaston, UK; c Massey University, Palmerston North, New Zealand; d Sawbridgeworth Medical Services, Jacobs & Gardens Neuro Centres, Sawbridgeworth, UK; e Birmingham Community Healthcare NHS Foundation Trust, Birmingham, UK; f School of Computing, University of Kent, Canterbury, UK; g Centre for Applied Psychology, University of Birmingham, Edgbaston, UK

**Keywords:** Disorders of consciousness, Neurodiagnostics, Disclosure, Sense-making, Interpretative phenomenological analysis

## Abstract

Functional neuroimaging and electrophysiological assessments can identify evidence of residual consciousness and cognition in patients with prolonged disorders of consciousness (PDOC) who are otherwise behaviourally unresponsive. These functional neurodiagnostics are increasingly available in clinical settings and are recommended by international clinical guidelines to reduce diagnostic and prognostic uncertainty, and thereby assist family caregivers in their best-interests decision-making. Nevertheless, little is known about how family caregivers make sense of the results of these state-of-the-art functional neurodiagnostics. By applying Interpretative Phenomenological Analysis (IPA) to interviews with family caregivers of patients with diagnoses of PDOC who had received a functional neurodiagnostic assessment, we identify three primary themes of sense-making: The special significance of “brain scans”; A dynamic sense-making process; Holding on to hope and holding on to the person. These themes highlight the challenges of helping family caregivers to balance the relative importance of functional neurodiagnostic results with other clinical assessments and identify an ability of family caregivers to hold a contradiction in which they hope for recovery but simultaneously express a rational understanding of evidence to the contrary. We offer several recommendations for the ways in which family caregivers can be better supported to make sense of the results of functional neurodiagnostics.

## Introduction

It is becoming increasingly clear that some patients with diagnoses of Prolonged Disorders of Consciousness (PDOC) nevertheless possess levels of consciousness and cognition that are not evident from their external behaviour. For example, some PDOC patients who fulfil the diagnostic criteria for unresponsive wakefulness syndrome (UWS; also known as vegetative state [VS]), who appear wakeful but show no behavioural evidence of awareness of themselves or their environments (Royal College of Physicians, 2020), have been found to exhibit brain activity indicative of, for example, speech comprehension, selective attention, and command-following (Claassen et al., 2021).

As the evidence base grows for the diagnostic and prognostic value of these brain imaging assessments of awareness – so-called functional neurodiagnostics (Schembs et al., 2021) – clinical guidelines in some countries now recommend their use in the PDOC diagnostic process when available (Giacino et al., 2018; Kondziella et al., 2020), while others, such as the UK (Royal College of Physicians, 2020), argue that their low sensitivity (Kondziella et al., 2016), among other challenges, warrants their use in the clinic *only* when the results can be interpreted and contextualized through dialogue between the research and clinical teams. An inevitable consequence of these recommendations is that the results of functional neurodiagnostics will contribute (and, indeed, are already contributing; see Boerwinkle et al., 2023) to profound best-interests decision-making processes. Consequently, it is vital to understand the ways in which decision-makers, such as family caregivers, interpret and use the information provided by these novel tools.

While the impact of caring for a relative with a diagnosis of PDOC is multifaceted, evidence suggests that family caregivers experience a range of psychological, social, and practical challenges to their quality of life, including psychological distress, deterioration in personal relationships, and disruption to daily life and occupation because of caregiving demands and financial pressures (Chinner et al., 2022). It is in this context, then, that family caregivers are required to contribute to best-interests decisions, including enduring decisions regarding withdrawing life-sustaining therapy. To make these decisions, family caregivers are presented with a range of clinical information, including the outcomes of behavioural assessments, occupational therapy and physiotherapy reports, and potentially the outcomes of functional neurodiagnostic assessments of so-called *covert* cognition and consciousness.

How then do family caregivers make sense of the results of functional neurodiagnostics? How do those results affect the family caregivers’ sense-making of their situation and their relationship with the patient, who may survive for many years after their injury?

Due to the considerable media attention surrounding high-profile case studies of positive results from functional neurodiagnostics, it is possible that such results are not weighted the same by family caregivers when compared with results from conventional diagnostic assessments, despite the latter being the clinical standard for diagnosis. Consequently, some authors argue that family caregivers are at risk of false hope or misinterpretation of the results (Samuel & Kitzinger, 2013). Uncertainty about patients’ states of consciousness has been described as causing a feeling of “Ambiguous Loss” in caregivers (Boss, 2007) which, coupled with potential false hope and misinterpretation due to “hype,” may obstruct their abilities to make reasoned decisions on behalf of the patients (Chinner et al., 2022).

### Current knowledge

In this context, three recent studies have investigated the impacts of functional neurodiagnostic results on family caregivers. Schembs et al. (2021) applied qualitative content analysis to interview data acquired from seven wives or mothers of patients with diagnoses of PDOC who had received a functional neurodiagnostic assessment as part of a research programme within the preceding 9 months. Their data exhibited a pattern of sense-making consistent with cognitive dissonance theory (Festinger, 1957), whereby family caregivers devalued or disregarded results that were inconsistent with prior expectations, while ascribing greater value to results that were consistent them. This interpretation suggests that functional neurodiagnostics in fact have little value for reducing caregiver uncertainty in the decision-making process and, given the significant financial cost of many functional neurodiagnostics, they should not be included in clinical assessments as family caregivers will only value results that confirm already held beliefs. However, due to the method of sampling, the above interview participants all had high levels of hope for recovery, and so may have stronger biases for seeking confirmation than other individuals across the breadth of experience of family caregivers of individuals with diagnoses of PDOC (Peterson, 2021). Consequently, the authors speculated that family caregivers’ epistemic beliefs may differentiate those who are more willing to adjust their beliefs about the patients’ diagnosis after receiving the results of a functional neurodiagnostic assessment, from those with less malleable beliefs (Kuehlmeyer et al., 2021). Furthermore, disclosure of the results of the functional neurodiagnostic to family caregivers was not standardized in this sample, with some participants not remembering the disclosure, suggesting that different impacts on sense-making and decision-making may be possible in contexts where functional neurodiagnostics are delivered as part of standard of care versus in a research context.

Relatedly, Peterson et al. (2021) provided a descriptive analysis of interviews with 12 family caregivers of patients with diagnoses of PDOC both before and after disclosure of the results of functional neurodiagnostic assessments performed as part of a research programme. This approach led Peterson et al. (2021) to identify a range of prior expectations and motivations for seeking out a functional neurodiagnostic assessment, including hopes to “correct” an existing diagnosis that was made on the basis of standard clinical methods, or to corroborate a caregiver’s belief that the patient was able to understand them when they speak. Following evidence of residual consciousness and cognition from a functional neurodiagnostic, some family caregivers described changes to the way they interacted with the patient because they felt that they had a greater understanding of the patient’s mental life. This information was also described as being valuable for encouraging clinical staff to similarly acknowledge a higher level of awareness than was evident in their behaviour. Consistent with data reported by Schembs et al. (2021), family caregivers were also described as devaluing or disregarding inconclusive results of the functional neurodiagnostics by, for example, suggesting that the patient moved too much during the assessment so that the data could not accurately reflect their brain activity. While the descriptive analysis from Peterson et al. (2021) is valuable, a structured qualitative analysis framework, taking into account underlying epistemological and ontological positions, may have supported the identification of divergent as well as convergent accounts across the interview data.

Recently, Boegle et al. (2022) applied reflexive thematic analysis to interviews conducted with nine family caregivers of patients with diagnoses of PDOC who were enrolled in a functional neurodiagnostic study while admitted to a neurorehabilitation centre. Echoing the interpretations of Schembs et al. (2021) and Peterson et al. (2021), caregivers similarly and, in at least one case explicitly, were biased towards accepting outcomes of functional neurodiagnostics that confirmed their prior beliefs: “You refuse to believe it, you only want to hear the good” (Boegle et al., 2022). Boegle et al. (2022) also highlighted the seeming contradiction of caregivers selectively accepting or disregarding neurodiagnostic outcomes depending on their prior beliefs while simultaneously expressing a desire to receive more information to clarify the patients’ condition.

Across these qualitative studies to date, it appears that family members tend to positively react when functional neurodiagnostic results confirm their pre-existing beliefs about the patient. However, when results are inconclusive, some tend to deny the results by questioning the validity of neuroimaging techniques (Boegle et al., 2022; Peterson et al., 2021; Schembs et al., 2021). On the other hand, those who have “accepted” the patient’s clinical diagnosis are more likely to accept inconclusive results (Peterson et al., 2021). Across studies, it is evident that pre-existing beliefs can affect how functional neurodiagnostic results are interpreted, though the method of disclosure may also affect this (Kuehlmeyer et al., 2021; Peterson, 2021).

### Our approach

Here, we sought to add to this limited body of work and address two limitations of the above literature. Specifically, all family caregivers in this study received a written report of the results as well as a meeting with research staff and/or clinical staff, thus ensuring a standardized disclosure process. Furthermore, we employed Interpretative Phenomenological Analysis (IPA; Smith et al., 2022) as our qualitative approach as its framework of phenomenology and hermeneutics is well-suited to exploring participants’ lived experience of critical life events and the way they give meaning to those events. IPA owns the interpretative role of the researcher in the process (Smith et al., 2022), who can be said to be engaging in a “double hermeneutic” in which they are making sense of the participant who is also making sense of their own experience. IPA draws from both a hermeneutics of *empathy* which tries as far as possible to gain an “insider account” of the experience and meanings, and a hermeneutics of *questioning* which attempts to use interpretation to illuminate that experience (Smith et al., 2022). The idiographic framework of IPA allows for a detailed focus on individual participant accounts and themes that represent both the commonalities and divergences between accounts. Taken together the theoretical underpinning of IPA supports an in-depth understanding of the potentially contradictory experiences and needs of family caregivers and therefore supports nuanced conclusions about how clinicians and researchers should frame the purpose and results of functional neurodiagnostics.

## Method

### Participants and recruitment

Purposive homogenous sampling was used to recruit participants who were family caregivers of patients with diagnoses of PDOC who had been enrolled on two electroencephalography (EEG) functional neurodiagnostic research studies in England. Ten family caregivers were first approached in person about this study by a member of their clinical team who, with their consent, passed their contact details to our research team to provide detailed study information. Six of the ten potential participants either decided to not participate or could not be reached to provide further study details. This led to three participants being recruited from the Bedside Test of Awareness for Disorders of Consciousness (BETADOC; Bareham et al., 2020) study’s cohort of personal consultees, and a fourth participant being recruited from the Language Processing After Trauma Cohort Study (LPAT; https://www.hra.nhs.uk/planning-and-improving-research/application-summaries/research-summaries/language-processing-after-trauma-lpat/) (Table 1). Small sample sizes are typical of IPA studies and align with IPA’s idiographic orientation and commitment to an in-depth account of individual lived experiences (Smith et al., 2022).

**Table 1.** Participant and interview details.

Pseudonym	Relationship to the person with diagnosis of PDOC	Aetiology	Time from injury to functional neurodiagnostic disclosure (months)	Time from disclosure to interview (months)	Length of interview (in minutes)
Rosa	Sister	Traumatic	8	7	38
Emma	Wife	Non-traumatic	22	25	17
Derek	Husband	Non-traumatic	12	26	19
Anna	Mother	Traumatic	27	27	45

### Functional neurodiagnostics

The BETADOC study involved longitudinal high-density EEG and behavioural assessments over 2-years while the LPAT study involved a single high-density EEG and behavioural assessment. Both studies investigated resting-state markers of residual functional networks, which are known to have diagnostic and prognostic value (Bareham et al., 2020). Additionally, the LPAT study investigated evidence for cognitive–motor dissociation (Cruse et al., 2012) and speech processing (Sokoliuk et al., 2021).

### Procedure for disclosure of research results

In the BETADOC study, a member of the research team and a member of the clinical team met with each family caregiver to provide written and verbal results disclosure. In the LPAT study, a member of the research team provided written and verbal feedback to a member of the clinical team who in turn disclosed the information to the family caregiver in a separate meeting. In both studies, family caregivers were provided with a written report to take home after the disclosure meeting.

### Data collection

Data were collected using semi-structured interviews conducted over the telephone by the third author (AC), a female student on a research Masters course. Participants were asked to be somewhere quiet where they could be alone for the interview. The interview schedule was developed by the research team and with guidance from a qualitative research support group consistent with guidelines for IPA methodology (Smith et al., 2022). It comprised open questions about how participants came to be involved in the study followed by more specific questions about their experience and sense-making in relation to the study procedure and the result disclosure. The interview schedule was used flexibly such that the researcher could follow up any unanticipated avenues that arose during the interview (Smith, 2017). As all participants received a copy of the interview schedule prior to consenting, our participants understood the interviewer’s goals and reasons for conducting the research. Each participant was interviewed on one occasion and interviews ranged between 17 and 45 min. Interviews were audio recorded using a digital voice recorder and transcribed verbatim for analysis. While participants communicated with the researcher by email and/or telephone prior to the interview as part of the consenting process, participants had no specific relationship with the interviewer. Participants were given the opportunity to review their transcript before analysis, with no participants suggesting any edits. No repeat interviews were carried out.

### Data analysis

The data were analyzed by the second and last author, the former a female student on a research Masters course and the latter a female qualitative researcher and clinical psychologist. The analysis process followed the steps reported by Smith et al. (2021). At the first step, the transcript for the first participant was read. The second step was making line-by-line descriptive, linguistic, and conceptual notes on a hard copy of the transcript. The third step was the development of experiential statements from initial notes which aimed to capture what is important to participants alongside how they make sense of these experiences (*claims and concerns*; Larkin et al., 2006). Steps four and five comprised identifying patterns across experiential statements which were clustered into personal experiential statements (PETs) and presented in a table. Consistent with the idiographic underpinning of IPA, this process was completed for one participant before moving on to the next. The final step was identifying commonalities and divergences among participant themes to develop a set of group experiential themes. The transcripts were cross-checked to ensure that the final set of themes were grounded in the data. Participants were not approached for feedback on the findings. The concept of data saturation is underpinned by a realistic ontology and therefore does not align with the assumptions of IPA. Instead, the researchers attended to the “quality indicators” for IPA studies, outlined by Nizza et al. (2021), focusing on developing a coherent and experientially rich account supported by participant quotations and interpretative commentary, and attending to convergence and divergence in participant accounts.

### Ethics

Participants recruited from the BETADOC consultee cohort were recruited under ethical approval provided by the University of Birmingham STEM Ethics Board. Participants recruited via the LPAT study were recruited under ethical approval provided by the West Midlands Coventry and Warwickshire Research Ethics Committee and the Health Research Authority, sponsored by the University of Birmingham. Eligible family caregivers were identified and approached by the respective clinical teams, who then signposted them to our research team.

All participants gave informed consent to participate. To reduce emotional distress from discussing sensitive topics, participants were sent the interview schedule before the interview so that they could decide whether they wanted to participate. Consent was verbally confirmed at the beginning of each interview. All audio recordings were destroyed upon completion of the transcription process. Anonymised transcripts will be stored under appropriate digital security for 10-years.

### Reflection

The first author (DC) has a background in cognitive neuroscience and has worked to develop functional neurodiagnostics for disorders of consciousness for 14-years. As part of that research, DC has frequent conversations with family caregivers of patients in PDOC, including disclosures of research results, and has previously worked with neuro-ethicists to argue the importance of disclosure (Graham et al., 2015). From DC’s experiences of disclosure discussions, he brought an anticipation that family caregivers will portray strong feelings of hope and biases in interpreting results. The second and third authors (KR and AC) were students on a research Masters course who did not have prior experience of PDOC personally or professionally. Their preconceptions were influenced by prevailing views within PDOC research and the media about biases in result interpretation. The final author (DV) is a qualitative researcher and clinical psychologist interested in how people make sense of their experiences. DV has worked clinically in neurorehabilitation and has encountered discourses within staff teams around false hope for families.

To avoid imposing their preconceptions on the data, the researchers had to be mindful of them and be alert to narratives that did not fit with their prevailing understanding. In addition to the steps to ensure rigour in data analysis outlined above, a reflective log was kept by KR, AC, and DV to scrutinize their own assumptions.

## Results

Three main themes are reported:

1. The special significance of “brain scans”

2. A dynamic sense-making process

3. Holding on to hope and holding on to the person

### The special significance of “brain scans”

A key feature across participants’ accounts was the sense that brain scans have the potential to provide unique knowledge about their loved one in the context of not having access to reliable information about their state of awareness. Participants described the impossibility of knowing about their family member’s mind state, which characterizes the situation of having a loved one with a PDOC. Emma explained: “um you just don’t, as I said before, you just don’t know. Once again, the brain’s so complicated no one really knows what’s going on up there at times but um [inhales]” (Emma, 109–113). Emma’s shift from *you* to *no one* suggests a dead end when it comes to knowing about what is happening for her husband. For Rosa, a key part of not knowing related to feeling unable to rely on information from medical professionals:

So we went through them telling us he’s not going to make it and they was going to turn his machines off and everything. Um but then we went to move him to a private room, he moved so they were saying you can’t be brain dead and make movements, so it doesn’t add up. (Rosa, 549–554)

Rosa’s lack of confidence in what she is told is captured in her assertion that *it doesn’t add up*.

In this context of not knowing, participation in the research became a source of hope for possible information and help for their loved one. For example, Rosa described:

Um I think the doctors were telling us they don’t know basically anything when it comes to his [her brother’s] mind state and his mind frame and, if he’s hearing or listening or if he’s awake or anything and, when I went through the research it kind of, was something that could kind of give us just a little bit o- of something or cause, I didn’t know how complicated the brain was. (Rosa, 58–63)

Here the absence of *anything* is contrasted with the research that has the potential to give *just a little bit o- of something*. The sense of hope for what the research can offer is also apparent in Derek’s description of his decision to participate in the research: “It [the research] didn’t disturb her, um so anything that might’ve been helpful in any treatment going forwards for [his wife] then I’ll grasp at anything” (Derek, 127–129). Derek’s use of *grasp* is echoed in Emma’s description: “Yeah, I think anyone who is approached [to participate] um obviously thinks oh there’s some lifeline here, we’ll grab it and see if something good will come out of it for the patient” (Emma, 38–40). Emma’s description of the research as a *lifeline* to *grab* conveys the significance that the research seems to take on for participants desperate to help their loved one. For Anna, the research is understood as part of her fighting for her son and as a way to understand what is happening for him. Her situation is complicated by initially being reliant on her husband and other son to share information with her:

they [her husband and son] don’t want to explain what happened for [son], and er I fighting for him and they told me n- something but I don’t understand nothing but when they done research and give me all the form I translate and I understand the situation my son he pass on. (Anna, 45–51)

Across the participant accounts, the special status given to the research in the context of participants’ desire to help their loved one but having few avenues for reliable information is conveyed.

In contrast to the unreliable and ambiguous knowledge about their loved one, for three of the participants the scans are understood as providing information that is concrete and definitive. For Derek there is a sense that the brain scans might provide conclusive knowledge about his wife’s state of health:

And um it [the research] was another form of finding out if there was any hope […] Yeah to see if there was progress in [wife’s] um let’s say state of health in er consideration of the brain, um and see where it went from there. (Derek, 40–45)

Emma also conveys what she understands as the definitive nature of the scans: “you, you hope that they may find something that, you know, there” (Emma, 78–79). Rosa expresses more explicitly the sense that the brain scans can provide direct access to her brother: “kinda offer like a, a look into, what his brain is kind of doing” (Rosa, 70–72). For her, there is a contrast between not knowing and the concrete nature of the scans that *actually connected* to her brother’s brain:

Because um I think it gave us – you know when you don’t know, you don’t know and he’s in the hospital and we’re like is he gonna make it, isn’t he, like is there anything? And they’re telling us they don’t know or, what they – they don’t know but they hope, but there’s hardly any chance that he’s going to. And then a test like that when it comes through even though it’s just a trial, it, it’s the only thing that we had that actually connected to his brain, just to see if it was doing anything you know. (Rosa, 83–90)

There is a sense that the brain scans can uniquely reconnect to the participant’s loved one and provide conclusive information about whether they are *there*.

For some participants, the research and perceived definitive nature of the disclosure are understood as important to guide their response to the situation. Derek explains what was important to him about participating in the research: “Well it would show that there was a reason to maintain hope or that this is it. The result of it all is gonna say this is [his wife] forever” (Derek, 191–198). Here Derek suggests that the disclosure can shape his emotional response to the situation in terms of maintaining hope. For Rosa also there is sense that the disclosure might be a way of readying herself emotionally to stay the course.

So you’re told one thing and you do your research and doing your research doesn’t help because sixty thousand people will say sixty thousand different things so you literally just taking each day as it comes and it got to a point where you just wanna know some things, like I need to know something. I can’t keep coming here every day, like, that’s when it starts messing with you. I’m coming here every day and I don’t know what to expect. (Rosa, 573–579)

There is a sense here of the emotional impact of the uncertainty of the situation. For Anna, the research seems to be understood as a way to legitimise her response of fighting for her son:

I prefer like everyone in situation like my son, been and done like every year scan for brain […] to see how much they develop them themselves, they how they improve because this helping a lot like if showing same stable, or you gonna fight and h- how you gonna continue your way you understand me? (Anna, 574–577)

Overall, this theme seems to suggest that the participants place special significance on the research as providing conclusive knowledge about their loved one in the context of not being able to directly access information about their loved one’s state of awareness while being desperate to do anything to help them. The significance of the research to participants is captured by Derek who, despite describing not being happy with the results, says “I’d do it again tomorrow” (Derek, 213).

### A dynamic sense-making process

Alongside descriptions of the results as definitive, participants articulated a dynamic process of sense-making in which their response to the results was influenced by a range of factors including their own felt sense about their loved one’s condition; their intimate knowledge of their loved one; and their readiness to receive the results. Participant accounts seemed to suggest that when the results corresponded with their understanding of their loved one’s state of awareness, they were able to integrate and accept even negative results. Rosa directly articulated the influence of expectations when she compared her own response to the results about her brother with that of her best friend:

My best friend was with me um and, like I said the information helped me, but it didn’t help her, cos her perspective of his [her brother’s] recovery was completely different. So personally, for me it helped me but for my best friend that was there with me for the whole journey, it kind of disheartened her. (Rosa, 188–193)

For Rosa, Derek, and Emma the results seemed to correspond with their expectations about their loved one’s state of awareness. Rosa described as helpful the feedback that her brother showed residual activity because it verified her own understanding about her brother’s condition: “I’ve just had my own picture of what was going on with him and, with the study, it kind of confirmed everything that I thought … and it helped real- it did help” (Rosa, 178–180). Derek and Emma were more equivocal about the usefulness of the results they received, but still articulated a sense of acceptance in relation to negative results: “Um well … it’s a bit what we expected to see. Not much improvement or no improvement” (Derek, 101–102). Derek’s modifier *a bit* hints at disappointment despite the results meeting his expectations. Emma, although “a little bit disappointed” (Emma, 105) by the negative results, seemed able to integrate these with her existing understanding of her husband’s state of awareness:

*Interviewer*: And did it [the feedback] change um how you thought about his condition?

*Emma*: No, not really because I think I has already accepted his position uh so it didn’t really change at all in my respect. (Emma, 236–237)

The responses of Rosa, Derek, and Emma can be contrasted to that of Anna when she received negative results about her son which contradicted her own understanding: “My son he try to explain for me I said that ‘no he’s not like this, you lying to me […]’. I crying. I want them see that he not he not like this” (Anna, 394–396). Here Anna conveys both her rejection of the results and her own intense emotional response to it.

Anna’s narrative highlighted how medical information was less important to her for understanding her son’s condition than her own knowledge of her son. She identified both the unique mother-and-son relationship (“Because y- you know, mum understand her child more than anyone.” Anna, 301–302) and the time spent with her son post-injury as fundamental sources of information about her son’s state of awareness: “[…] I want see them, let them see how I see my son because everyone, not like, doctor he can’t see what I see, because I been there every day […]” (Anna, 126–129). For Anna, the first set of results that did not fit with her felt experience were disregarded while feedback from a second EEG indicating a positive result was readily accepted and integrated into her own understanding of her son’s condition:

First scan I translated all in English and said no that’s not my son what they talking about, and after they done second research and I said yeah this one, this one is what I talking about. Um he improve, he started showing you know. (Anna, 168–172)

Rosa explicitly articulated the dynamic rather than static process of sense-making in relation to the disclosure of results. Her narrative introduced the concept of readiness as important for her ability to engage with the feedback (“and if you are ready for the information as well,” Rosa, 485). For Rosa, readiness seemed to relate to her emotional state:

Um [inhales], emotionally I think, I think I got strong – not stronger, I wouldn’t say stronger but – oh how would I describe? I think at first I was just like I wasn’t, when I say I wasn’t ready, I was just an emotional mess. I didn’t have a clue whether I was coming or going and then I had like sixty thousand different things to focus on my brain was everywhere (Rosa, 598–605)

At the same time, Rosa described her openness to the feedback as part of her preferred approach to managing challenges: “I’d rather be hit with it like just tell me exactly what it is, if it was to say he had no brain activity or nothing wasn’t working that’s what it is. […] I can prepare myself then” (Rosa, 470–472), contrasting her own approach with that of her friend: “she kind of likes to live in denial” (Rosa, 512).

This theme highlights that participants used a range of sources of information to make sense of the results disclosure. These factors can be understood as going beyond expectations to include participants’ felt sense and intimate knowledge of their loved one based on their unique relationship with and knowledge of their loved one as well as personal coping styles and feeling emotionally prepared. Sense-making therefore seems to be an emotional as well as cognitive process.

### Holding on to hope and holding on to the person

Across all participant accounts, hope seemed to be fundamental to the unique experience of having a loved one with a PDOC, as articulated by Derek: “Well, in [wife’s] situation, you live in hope” (line 38). At the same time, the narratives of three participants distinguished hope from their expectations for their loved one’s recovery. Emma described her anticipation of the disclosure: “but I think you know the person that – the loved one that’s been, you know, assessed, and I think probably deep down you know what reactions will become but, you’ve always got that hope. Always” (Emma, 41–44). Here Emma’s repetition of *always* conveys the unwavering nature of hope which is different from her expectations for the results. Describing his response to negative results, Derek similarly distinguished what he *anticipated* for the results from his continued hope:

It didn’t, it didn’t really supply me for any more than I’d, I’d anticipated in [wife’s] state of health so I wasn’t disappointed with it. At the same time, I wasn’t happy with it you know [laughs]. I was, I’m always living in hope here. (Derek, 112–118)

The shift in tense from past tense “I was” to present “I’m” hints that for Derek, like Emma, the negative results did not alter his fundamental state of hope.

In her narrative, Rosa explicitly differentiates hope from denial:

Like some people just prefer to live in denial and, you know, it’s not that I don’t have as much hope as her [Rosa’s friend] but I try to be realistic about the situation so when you get some information given to you […] I was ready to receive it. (Rosa, 515–519)

Here Rosa articulates that it is possible to hold hope while remaining *realistic* and open to information (Rosa, 670). Across these participants there is a sense that hope can be separated from, and held alongside, their (realistic) beliefs and knowledge about their loved one’s recovery. Rosa summarized this: “Be realistic, prepare for the worst and hope for the best. Literally” (Rosa, 630–635).

The participants’ narratives help to make sense of what seems to be the transcendent nature of hope. Specifically, the act of holding onto hope seemed to be central to the participants’ continued relationship with and emotional connection to their loved one. Rosa explains:

Because it w- it was literally it, it’s hope you know. And in the hospital you tend to find that they try and- they prepare you for the worst but sometimes y- you know you- I, I understand exactly what’s going on in the situation [interviewer: yeah] but that’s somebody you love and you wanna have the hope, you know, don’t take it away. And you feel like in the hospital journey it- they kind of take it away from you. (Rosa, 357–364)

Rosa’s phrase *because that’s somebody you love* explains the need to maintain hope while she *understand[s] exactly what’s going on in the situation*. For Emma, maintaining hope seemed to be an act that signified not giving up on her husband. She described her motivation to participate in the research as an act of hope:

At least, they- you, you tried, you done, you tried, you done your best to see if there’s anything there that would help [interviewer: yeah]. That’s the only, I think if you don’t give someone the chance then, you don’t know there’s always a maybe, a maybe or if only. (Emma, 128–134)

This perhaps explains Emma’s statement: “And I don’t think you should ever give up any hope whatsoever” (Emma, 46–47). The sense of not giving up is reflected in Anna’s account of her response to the results:

Im fighting, I’m, I’m, I’m not go- I said that I’m gonna fight for him. Show them different from what they said [Interviewer: OK] in the beginning and second one he improve and I told you all positive thing count. I- I said for myself I show them [son] gonna come back to me. (Anna, 213–220)

This theme suggests that the act of holding on to hope is fundamental to participants to maintain an emotional connection with and not give up on their loved one. This understanding helps to shed light on the ways that participants make sense of the results, maintaining hope in the face of (for Derek, Emma and Rosa) or rejecting (for Anna) negative results.

## Discussion

With the growing availability and recommended use of functional neurodiagnostics in assessment of PDOC, it is vital to understand the ways in which family caregivers make sense of the results of these tools. By applying Interpretative Phenomenological Analysis to interview data, we identified three main themes that provide valuable insights into caregivers’ sense-making processes, and hint at ways in which functional neurodiagnostics can be more usefully incorporated into the best-interests decision-making process.

First, our theme of “The special significance of ‘brain scans’” reflects the views of our participants that functional neurodiagnostics uniquely connect to patients’ mental states and provide conclusive information, beyond that provided by the standard clinical behavioural diagnostic approaches. Indeed, the high value ascribed to functional neurodiagnostics is evident from Derek who stated that he would “do it again tomorrow,” despite his describing a not overly positive experience of the assessment period itself. This view is in contrast with the views of the caregivers interviewed by Schembs et al. (2021) who ascribed functional neurodiagnostics with a relatively lower subjective significance, with several participants even forgetting whether they had happened. As Schembs et al. (2021) argue, it is possible that this disparity arises because, in their study, the neurodiagnostics were part of routine care rather than a separate research project, such as with our participants. This may have made the neurodiagnostic experience of our participants more of “an event,” rather than just another of the many diagnostic assessments that patients in PDOC receive. This view is espoused by Peterson (2021) who argues that, when neurodiagnostic results are provided holistically alongside other assessment results, caregivers may not recognize their significance.

This theme highlights a difficult balance for clinical and research teams in how functional neurodiagnostics are framed and how results are delivered. Indeed, functional neurodiagnostics have been described as “concrete measurable signs of awareness” and presented in a seemingly infallible light (Lewis et al., 2023; Scolding et al., 2021). While functional neurodiagnostics have certainly provided valuable information beyond conventional approaches (Egbebike et al., 2022), their well-documented poor diagnostic and prognostic sensitivity (Kondziella et al., 2016) should make us wary of setting these methods as a gold-standard that outweighs the combined evidence of conventional assessments. Rather, in the absence of a highly sensitive gold-standard functional neurodiagnostic, the goal should be to incorporate them into the wider assessment process and support family caregivers to appropriately weight their evidence alongside other sources, both positive and negative (see Boerwinkle et al., 2023, for one framework for incorporating into the clinical pipeline). Indeed, several authors of the UK guidelines for PDOC argue that assessments should place less emphasis on whether the patient is conscious or not, and more on “what the individual can do” and their likely trajectory, as this is key to best-interests decision-making (Gill-Thwaites et al., 2023) and requires input from multidisciplinary sources.

In the context of the special significance assigned by family caregivers to functional neurodiagnostics delivered as part of a research study (see also Peterson et al., 2021), Jox et al. (2012) caution that neurodiagnostic results may be viewed as “ultimate proof.” Our second theme suggests a more “dynamic sense-making process” in which family caregivers draw on, and are influenced by, a range of sources when making sense of the results. Schembs et al. (2021), for example, observe that in their sample of highly optimistic family caregivers, positive results were accepted and negative/null results disregarded, as a means of minimizing cognitive dissonance. Peterson et al. (2021) also observed that family caregivers only questioned the validity of negative/null results, while acceptance was closely tied to caregivers’ expectations. Similarly, here Anna takes us along precisely this cognitive dissonance path to sense-making by describing how she disregarded the negative results of a first assessment but subsequently accepted the positive results of a second assessment.

Our results further build on these observations of expectation bias by identifying additional sources of information that support family caregivers’ sense-making processes. Anna, for example, describes the overarching value of her own unique relationship with her son, which, for her, outweighed evidence from any other source – “Mum understand her child more than anyone” (Anna, 301–302). While Boegle et al. (2022) argue that clinicians should provide family caregivers with more scientific information to counteract interpretations that are biased by prior expectations, Anna’s experience suggests that this approach is unlikely to always achieve the desired outcome.

Within this theme of dynamic sense-making, Rosa also highlighted the importance of preparedness for receiving results of neurodiagnostics, with her emotional state influencing her sense-making. This is consistent with Peterson et al.’s (2021) call for pre-disclosure discussions with family caregivers, which may help identify existing expectations while supporting family caregivers in achieving a state of preparedness to receive the results.

Finally, our third theme “Holding on to hope and holding on to the person” reflects the fundamental nature of hope within the experiences of our family caregivers. Indeed, holding on to hope appeared to be impervious to any information provided by the functional neurodiagnostics. One interpretation of this would be in-line with the view that family caregivers are biased to only accept results that fit with their prior expectations (Boegle et al., 2022; Peterson et al., 2021; Schembs et al., 2021). However, we find here that family caregivers can hold a rational understanding of a negative result and a realistic expectation of diagnosis and prognosis while simultaneously holding on to hope. It is as if holding on to hope is central to the family caregiver’s relationship with the patient and a part of doing right by them, because “that’s somebody you love” (Rosa, 357–364). This apparent holding on to a contradiction is key to understanding the sense-making of family caregivers. Indeed, some authors argue that providing family caregivers with more evidence and a clearer understanding of the import of functional neurodiagnostics will mitigate false hope or false despair (Boegle et al., 2022; Peterson et al., 2021). Schembs et al. (2021) also concluded that family caregivers are “protecting their hope” by disregarding negative results. However, our results indicate that hope may be an unshakable facet of some family caregivers’ experiences that doesn’t necessarily need to affect, or be affected by, their ability to be realistic in the face of diagnostic and prognostic evidence. Indeed, Peterson et al. (2021) observed that their family caregivers were, on the whole, able to understand the results and implications of their functional neurodiagnostics. Hope, then, need not be a condition to be pathologised and mitigated in the service of best-interests decisions.

This seeming contradiction of hope alongside acceptance is reminiscent of the proposition from the literature on Ambiguous Loss in which family caregivers may benefit from being able to “hold a paradox” about a patient who is simultaneously there and not (Boss, 2010). Indeed, Ambiguous Loss is precisely the situation described by family caregivers of patients with diagnoses of PDOC – the feeling of loss of a loved one who is nevertheless physically present (Boss, 2007; Soeterik et al., 2018). In their description of Ambiguous Loss, Boss (2010) specifically argues that “to stay strong, people *need hope* despite ambiguous loss.” The unshakable hope of our family caregivers may reflect such a mechanism of staying strong. Rather than attempting to remove hope, in the belief that it will result in more rational decision-making, clinicians and researchers may instead acknowledge family caregivers’ hope and support them in balancing their unique relationship to the patient with other sources of hope to be found in life beyond that relationship (Boss, 2010; Giovannetti et al., 2015).

### Limitations

As our participants were those who had also consented on behalf of the patient to take part in a functional neurodiagnostic research study, our sample may have been biased towards those most invested in functional neurodiagnostic disclosure (see theme: The special significance of “brain scans”). Furthermore, as all patients were recruited from an active neurorehabilitation context, our family caregiver sample may have been biased towards those more hopeful for recovery (see theme: Holding on to hope and holding on to the person; see also Kuehlmeyer et al., 2021). Similarly, our sample were recruited from the UK’s health system. However, there are significant differences between countries in the legal and healthcare funding frameworks for PDOC, which will inevitably impact upon family caregivers’ attitudes and expectations towards functional neurodiagnostic results (Maurer-Karattup et al., 2022).

Our initial aim for this study was to conduct interviews with family caregivers soon after disclosure, as we achieved with Rosa. However, the COVID-19 pandemic struck early in our recruitment period and required that we cease all clinical research, thus introducing significant delays for some participants. Consequently, as a longer time had elapsed between functional neurodiagnostic disclosure and our interviews for 3-participants (∼2-years) their accounts may be considered retrospective and so may not reflect their initial responses and sense-making at the time of disclosure.

Finally, while the idiographic underpinning of IPA means that it is designed to work well with small samples of participants with shared experiences, it must be acknowledged that our interviews varied in length, with two shorter interviews. However, all interviews provided rich data in relation to the research question with all themes exemplified throughout by quotations. Future research following participants through the initial stages after the disclosure interview (see Peterson et al., 2021) alongside a structured qualitative analysis framework, such as IPA, will enrich our understanding of the nuanced responses of family caregivers.

### Recommendations

By combining our results with those from the currently limited literature of functional neurodiagnostic disclosure in PDOC, we offer several recommendations that we hope will benefit clinical and research practice and the experiences of family caregivers:

#### Communicate clearly that functional neurodiagnostics are both special and not

In light of evidence that family caregivers may only accept results if they match with existing expectations, Schembs et al. (2021) questioned whether functional neurodiagnostics have any value at all in the PDOC diagnostic process, especially given their high cost. Our results indicate that, while in some cases strongly held beliefs can trump diagnostic evidence, family caregivers are capable of understanding and accepting evidence from a range of sources, including functional neurodiagnostics. Consequently, these assessments do have value for best-interests decisions. Furthermore, we and others have argued for the ethical imperative to disclose the results of functional neurodiagnostics when they are available (Graham et al., 2015).

However, from our results, and those of others (Boegle et al., 2022; Peterson et al., 2021; Schembs et al., 2021), it is clear that some family caregivers ascribe greater value to the results of functional neurodiagnostics than to other sources of information. It is also the case that many researchers and clinicians do the same when communicating results (Samuel & Kitzinger, 2013). Such high levels of assigned value likely derive from the view that they reveal truths about the patient that are “hidden” from outward view. However, a critical eye on these assessments reveals their currently low sensitivity (Kondziella et al., 2016). Consequently, we recommend that family caregivers will benefit from being provided with a balanced understanding of both the sensitivity issues of functional neurodiagnostics alongside their potentially added value, all delivered as part of a wider holistic description of “what the patient can do” (Gill-Thwaites et al., 2023) accumulated across multidisciplinary sources.

#### Increasing family caregiver preparedness

Our results indicate that outcomes of functional neurodiagnostics can be experienced differently depending on the emotional preparedness of the family caregiver. One way to help family caregivers understand the relative value of functional neurodiagnostics then, is to ensure that pre-disclosure discussions appropriately contextualize the approach and help family caregivers to prepare for the results and the potentially strong emotional reactions they can elicit. Indeed, a frequent dialogical approach is standard for best-interests discussions in neurorehabilitation of PDOC in the UK (Royal College of Physicians, 2020), and would therefore provide an existing framework within which to situate discussions of functional neurodiagnostics alongside discussions of behavioural assessments, physiotherapy assessments etc.

#### Draw on the dementia literature to support family caregivers

Peterson et al. (2021) highlight the value of the existing literature on Alzheimer’s Disease, for example, which provides a framework for disclosing results in a way that minimizes misunderstandings and distress. Similarly, the contradictory experiences of some of our participants, who hold both hope and understanding, suggests that we may further support family caregivers by considering the literature regarding Ambiguous Loss and Latent Grief in family caregivers of people with dementia. Indeed, many patient characteristics overlap between late-stage dementia and PDOC – i.e., a state of being present but not. Furthermore, many patients with PDOC may ultimately share long-term care facilities with those with late-stage dementia, thus providing relevant and overlapping experience in the clinical teams.

#### Recognize hope without pathologising

Our results suggest that hope, specifically, can be held simultaneously with a realistic and rational understanding of a patient’s likelihood for recovery. Indeed, family caregivers of patients with PDOC will experience a range of emotions and should be supported to recognize them as normal responses to a traumatic situation (Boss, 2010; Kitzinger & Kitzinger, 2014). Consequently, clinicians and researchers should be made aware that hope may not be a situation to be avoided in the face of negative results, or to be reasoned away, but rather is one to be redirected in balancing their Ambiguous Loss (Boss, 2010).

### Conclusions

As functional diagnostics for PDOC become more accessible, both clinicians and researchers will increasingly be required to disclose results to family caregivers in a way that minimizes misunderstanding and supports best-interests decision-making. Our interviews with family caregivers here highlight the challenges inherent in communicating the relative value of functional neurodiagnostics and identify an apparent ability of family caregivers to both hope for recovery and simultaneously comprehend conflicting diagnostic evidence. By understanding family caregiver sense-making of functional neurodiagnostics, and supporting them in holding any contradictions, we will ensure more appropriate decision-making and minimize caregiver burden.

## Data Availability

To protect participant and patient confidentiality, interview transcripts are only available upon reasonable request to the corresponding author.
